# Phosphorylation within Intrinsic Disordered Region Discriminates Histone Variant macroH2A1 Splicing Isoforms—macroH2A1.1 and macroH2A1.2

**DOI:** 10.3390/biology10070659

**Published:** 2021-07-13

**Authors:** Sebastiano Giallongo, Oriana Lo Re, Gabriela Lochmanová, Luca Parca, Francesco Petrizzelli, Zbyněk Zdráhal, Tommaso Mazza, Manlio Vinciguerra

**Affiliations:** 1 International Clinical Research Center, St’Anne University Hospital, Pekařská 53, 65691 Brno, Czech Republic; sebastiano.giallongo@fnusa.cz (S.G.); oriana.lore@fnusa.cz (O.L.R.); 2 Department of Biology, Faculty of Medicine, Masaryk University, Kamenice 753/5, 62500 Brno, Czech Republic; 3 Department of Translational Stem Cell Biology, Medical University of Varna, 9002 Varna, Bulgaria; 4 Mendel Centre for Plant Genomics and Proteomics, Central European Institute of Technology, Masaryk University, Kamenice 753/5, 62500 Brno, Czech Republic; gabriela.lochmanova@ceitec.muni.cz (G.L.); zdrahal@sci.muni.cz (Z.Z.); 5 Laboratory of Functional Genomics and Proteomics, National Centre for Biomolecular Research, Faculty of Science, Masaryk University, Kamenice 753/5, 62500 Brno, Czech Republic; 6 IRCCS Casa Sollievo della Sofferenza, Bioinformatics Unit, Viale Cappuccini 1, 71013 San Giovanni Rotondo, Italy; l.parca@css-mendel.it (L.P.); f.petrizzelli@css-mendel.it (F.P.); t.mazza@css-mendel.it (T.M.); 7 Department of Molecular Medicine, Sapienza University of Rome, Viale Regina Elena 291, 00161 Rome, Italy

**Keywords:** macroH2A1, mass spectrometry, post-translational modifications

## Abstract

**Simple Summary:**

MacroH2A1, a histone H2A variant, is present as two alternative splicing isoforms, macroH2A1.1 and macroH2A1.2, which are finely regulated through several mechanisms, including post-translational modifications (PTM). In this article, the authors provide the PTM pattern of macroH2A1.1 and macroH2A1.2 in the same experimental setting through mass spec analysis. They report a different phosphorylation level in their intrinsically disordered linker region, which can be responsible for their different biological role, as computational analysis shows.

**Abstract:**

**Background:** Gene expression in eukaryotic cells can be governed by histone variants, which replace replication-coupled histones, conferring unique chromatin properties. MacroH2A1 is a histone H2A variant containing a domain highly similar to H2A and a large non-histone (macro) domain. MacroH2A1, in turn, is present in two alternatively exon-spliced isoforms: macroH2A1.1 and macroH2A1.2, which regulate cell plasticity and proliferation in a remarkably distinct manner. The N-terminal and the C-terminal tails of H2A histones stem from the nucleosome core structure and can be target sites for several post-translational modifications (PTMs). MacroH2A1.1 and macroH2A1.2 isoforms differ only in a few amino acids and their ability to bind NAD-derived metabolites, a property allegedly conferring their different functions in vivo. Some of the modifications on the macroH2A1 variant have been identified, such as phosphorylation (T129, S138) and methylation (K18, K123, K239). However, no study to our knowledge has analyzed extensively, and in parallel, the PTM pattern of macroH2A1.1 and macroH2A1.2 in the same experimental setting, which could facilitate the understanding of their distinct biological functions in health and disease. **Methods:** We used a mass spectrometry-based approach to identify the sites for phosphorylation, acetylation, and methylation in green fluorescent protein (GFP)-tagged macroH2A1.1 and macroH2A1.2 expressed in human hepatoma cells. The impact of selected PTMs on macroH2A1.1 and macroH2A1.2 structure and function are demonstrated using computational analyses. **Results:** We identified K7 as a new acetylation site in both macroH2A1 isoforms. Quantitative comparison of histone marks between the two isoforms revealed significant differences in the levels of phosphorylated T129 and S170. Our computational analysis provided evidence that the phosphorylation status in the intrinsically disordered linker region in macroH2A1 isoforms might represent a key regulatory element contributing to their distinct biological responses. **Conclusions:** Taken together, our results report different PTMs on the two macroH2A1 splicing isoforms as responsible for their distinct features and distribution in the cell.

## 1. Introduction

Chromatin compaction regulates gene expression in all eukaryotic cells. Canonical histones (H1, H2A, H2B, H3, and H4) can be substituted by histone variants in a subset of nucleosomes, changing the structure and the function of chromatin domains [1]. Unique patterns of genomic distribution have been described for histone variants. They can be deposited and removed by *ad hoc* molecular machineries, and play fundamental roles in the first stages of embryonic development, in the lineage commitment of stem cells and the reverse phenomenon of somatic cell reprogramming to pluripotent stem cells [1]. Variants of core histone H2A are numerous and are involved in multiple processes. H2A.X regulates DNA damage repair [1]. H2A.Z regulates chromosome segregation [1]. H2A.BBD is enriched in active chromatin domain [1]. MacroH2A1 is a H2A found only in vertebrates; it encompasses a histone domain that is highly similar to H2A and a non-histone domain of large size, called “macro” domain. In fact, macroH2A1 is the largest H2A variant, and it displays in two alternatively exon-spliced isoforms: macroH2A1.1 and macroH2A1.2. These two isotypes are quite identical with the exception a 30-amino-acid region in the macro domain. MacroH2A1 isotypes are abundant on the facultative heterochromatin (such as the one of the inactive X chromosome) and regulate cell plasticity during stemness and carcinogenesis [2]. MacroH2A1 proteins were originally reported as able to substitute canonical H2A in approximately 3% of all nucleosomes pool [3]. MacroH2A1 proteins can also be liberated in the extracellular space upon cell death, and serve as circulating disease biomarkers [4]. Furthermore, macroH2A1 isoforms participate in the formation of senescence-associated heterochromatic foci (SAHF) in cells undergoing senescence [2]. H2A-H2B dimers can be removed and exchanged more easily than the stable H3-H4 core, within the nucleosome structure [5]. The N-terminal histone tails of all histones and the C-terminal tails of H2A histones stem from the nucleosome core structure. These tails are preferentially accessible and target sites for many post-translational modifications (PTMs) [6]. H2A and H2A variants share some PTMs; however, many modifications characterize specifically histone variants [7]. Moreover, macroH2A1.1 and macroH2A1.2 have unique roles in controlling cell plasticity and proliferation [2,8,9,10,11]. In fact, these two isoforms diverge only for a few amino acid residues and for their capacity to bind ADP-ribose, a property that might explain their specific functions documented so far [2,8,9,10,12,13]. Some PTMs on macroH2A1 variant histones have been reported, such as phosphorylation (T129, S138) and methylation (K18, K123, K239) [7]. Monoubiquitination on K116 has been instead reported for macroH2A1.2 [14]. Other common PTMs, such as acetylation, have never been identified on macroH2A1 isoforms, and their roles remain elusive. No study to our knowledge has analyzed extensively, and in parallel, the PTM of macroH2A1.1 and macroH2A1.2 in the same experimental setting. This information could facilitate the understanding of their distinct biological functions in health and disease.

In the present study, we used a mass spectrometry-based approach to examine the sites for phosphorylation, acetylation, and methylation in green fluorescent protein (GFP)-tagged macroH2A1.1 and macroH2A1.2. For the first time, we show a quantitative comparison of histone marks between the two isoforms. Furthermore, our computational analyses offer new insights on the consequences of selected PTM on macroH2A1.1 and macroH2A1.2 structure and function. Altogether, our results provide evidence that the phosphorylation status in intrinsically disordered linker region in macroH2A1 proteins provides a key regulatory element, which in context with isoform-specific domain mediates distinct biological response.

## 2. Materials and Methods

### 2.1. Cell Cultures

The HepG2 parental cell line (ATCC) was cultured in DMEM (1X) supplemented with 10% fetal bovine serum (FBS) and 1% penicillin/streptomycin. Stable overexpression of a macroH2A1.1-GFP or macroH2A1.2-GFP transgenes/fusion proteins was achieved by lentiviral transduction, as previously described [15,16,17].

### 2.2. Immunoaffinity Enrichment of (GFP)-Tagged macroH2A1.1 or macroH2A1.2 Isoforms for Mass Spectrometric Analysis

Transfected HepG2 cells were lysed with RIPA buffer supplemented with Phosphatase Inhibitor Cocktail 2 (#P5726, Sigma-Aldrich, Prague, Czech Republic), 1 mM PMSF, and 45 mM sodium butyrate. (GFP)-tagged macroH2A1.1 and macroH2A1.2 fusion proteins were immunoprecipitated using ChromoTek GFP-Trap^®^ (#gtma-20, ChromoTek, Planegg-Martinsried, Germany) according to manufacturer instructions. The immunocomplex was washed five times with 500 μL of 50 mM ammonium bicarbonate buffer (ABC). The beads were divided into two fractions. Protein in fraction 1 was subjected to on-bead digestion using 20 ng·μL^−1^ trypsin (sequencing grade modified, Promega Corporation, Fitchburg, WI, USA) in 50 μL of 50 mM ABC. The digestion was performed for 4 h in thermomixer at 37 °C and 1100 rpm, the beads were magnetically separated, and the sample was incubated in thermomixer overnight at 37 °C and 750 rpm. Protein in fraction 2 was subjected to on-bead derivatization of lysine residues before enzymatic digestion. The beads were resuspended with 100 mM ABC to a final volume of 30 μL, and 0.5 μL of NH_4_OH was added. A 10 μL portion of propionylation reagent, freshly prepared by mixing propionic anhydride and acetonitrile in a 1:3 ratio, was immediately added to the sample. The pH was adjusted to 8 by NH_4_OH, then the sample was incubated for 20 min in a thermomixer at 37 °C, 700 rpm. The beads were magnetically separated, the sample was washed three times with 200 μL of 100 mM ABC, and the second round of propionylation was carried out with the same protocol. After derivatization, on-bead protein digestion was performed using 30 ng·μL^−1^ trypsin in 30 μL of 100 mM ABC. Following overnight incubation at 37 °C, the beads were magnetically separated, and the digest was collected. Fractions 1 and 2 were filtered through Ultrafree-MC LH Centrifugal Filter 0.45 μM (UFC30LH25; Merck, Darmstadt, Germany), the samples were acidified, and the sample volume was reduced in a Savant SPD121P concentrator to 10 μL (Thermo Fisher Scientific Inc., Waltham, MA, USA).

### 2.3. Mass Spectrometry, Database Searches, and Quantification of Peptide Forms

Tryptic digests of (GFP)-tagged macroH2A1.1 and macroH2A1.2 fusion proteins, each represented by four biological replicates, were measured using liquid chromatography tandem mass spectrometry (LC-MS/MS), as previously described [18]. The RAW mass spectrometric data files were analyzed using Proteome Discoverer software (Thermo Fisher Scientific, Waltham, Massachusetts, USA; version 2.2) with an in-house installation of Mascot (Matrixscience, London, England, U.K.; version 2.6.2) to compare acquired spectra with entries in the UniProtKB human protein database (version 2018_09; 21053 protein sequences), modified cRAP contaminant database (based on http://www.thegpm.org/crap/, version 2018 11; 112 sequences) and in-house macroH2A1 database (version 2018_11; 6 protein sequences). For database searches trypsin enzyme specificity with up to six missed enzyme cleavages was used and other parameters were set as previously [18]. For database searches of chemically derivatized samples, propionylation (K) was added. Values of precursor areasof macroH2A1.1 and macroH2A1.2 peptides were log_2_-transformed and normalized to the sum of selected non-modified peptides. Mean and standard deviation were calculated for each peptide form. Differences between samples in normalized peptide abundances ≥1.5-fold were considered as significant, and the significance of differences was assessed using Student’s *t*-tests, setting the significance threshold at *p* < 0.05.

### 2.4. Immunoblotting Analyses

Upon immunoprecipitation of (GFP)-tagged macroH2A1.1 and macroH2A1.2 fusion proteins using GFP-Trap® Magnetic agarose kit (ChromoTek, Planegg-Martinsried, Germany), immunoblotting analyses were performed as described previously [17]. Primary antibodies were obtained from Activ Motif (Waterloo, Belgium; macroH2A1.1 and macroH2A1.2).

### 2.5. In-Silico Structural Analyses

The primary sequences of macroH2A1.1 and macroH2A1.2 were downloaded from Uniprot (entry O75367-2 and O75367-1, respectively). Local sequence alignments were performed with BlastP [19] used with default parameters.

The PDB structures 1zr3 and 1zr5 of macroH2A1.1 and macroH2A1.2 were downloaded from the Protein Data Bank [20]. Moreover, the structures of macroH2A1.1 bound to ADP-ribose and macroH2A represented by a small peptide bound to SPOP (speckle-type POZ protein) were retrieved as 3iid and 3hqh, respectively. The structural visualization and general analysis (e.g., the distance between the alpha-carbon atoms of different residues and structural alignment) were performed using UCSF Chimera [21].

Prediction of kinase-specific events was performed with AKID [22]. It is a tool able to predict kinase-specific phosphorylations using the target peptide sequence and key residues (called determinants of specificity). This tool has been trained on the human kinome and was shown to predict kinase-specific events for 496 human kinases. Only high-score (>0.8) predictions were considered in this study.

Inference of nucleotide-binding sites was performed using Nucleos [23,24]. It is a tool able to infer nucleotide-binding sites by predicting the binding sites of its components (e.g., nucleobase, carbohydrate, and phosphate) and then combining them into different types of nucleotide-binding sites. It has been run with default parameters.

## 3. Results

### 3.1. Expression and Purification of GFP-Tagged macroH2A1.1 and macroH2A1.2 in Hepatoma Cells

**Figure 1:**
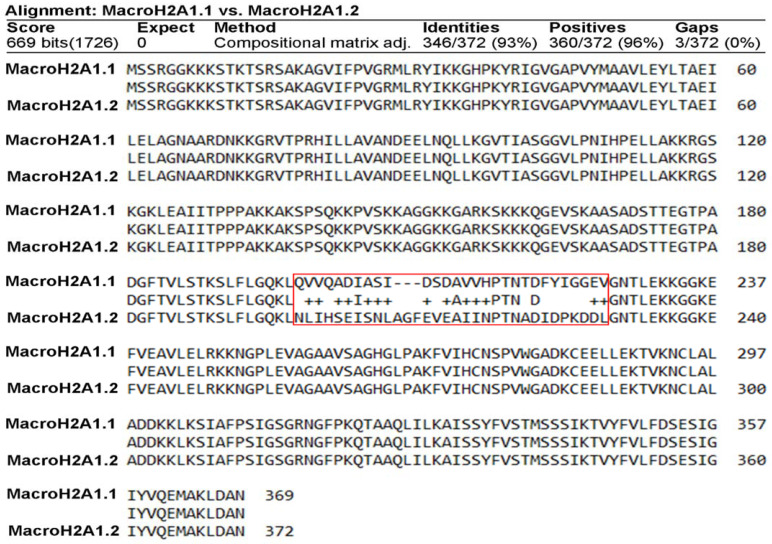
Sequence alignments of macroH2A1.1 versus macroH2A1.2 (human). Local alignment of the sequences of macroH2A1.1 and macroH2A1.2 performed with BlastP.

**Figure 2:**
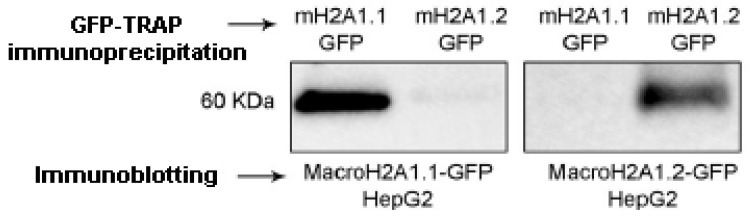
Immunoblotting analysis to confirm macroH2A1 splicing isoforms isolation from the fraction eluted from HepG2 overexpressing macroH2A1.1-GFP (mH2A1.1-GFP) and macroH2A1.2-GFP (mH2A1.2-GFP) following immunoprecipitation with GFP-Trap®.

The two exon splicing isoforms of macroH2A1, macroH2A1.1, and macroH2A1.2 are identical except for a 30-amino-acid region in the macro domain (Figure 1). This domain in macroH2A1.1 binds NAD-derived metabolites, such as ADP-ribose, whereas macroH2A1.2 does not [2,8,9,10,12,13], a structural feature that has been solved at the crystal level [25,26]. It has been proposed that this unusual domain structure of macroH2A integrates independent functions that are instrumental in establishing a functionally unique chromatin domain, together with features related to its histone-fold domain [27]. In addition, the tails and regions outside of the histone fold of macroH2A1.1/macroH2A1.2 may contribute as well to chromatin regulation through PTMs. It is thus of critical relevance to isolate macroH2A1.1 and macroH2A1.2, separately and in parallel, to study their PTMs. To achieve this aim, we ectopically overexpressed them as GFP-tag proteins, separately, in HepG2 hepatoma cells using lentiviral transduction to obtain macroH2A1.1-GFP and macroH2A1.2-GFP overexpressing cell lines [15,16,17,28]. MacroH2A1.1-GFP or macroH2A1.2-GFP fusion proteins were isolated from their respective HepG2 cell line using GFP-Trap® Magnetic agarose Kit. This tool is composed of anti-GFP nanobody/VHH bound to magnetic agarose beads. To confirm macroH2A1.1-GFP or macroH2A1.2-GFP immunoprecipitation, immunoblotting analysis was performed on the fraction eluted from the beads. Results confirmed macroH2A1.1-GFP isolation or macroH2A1.2-GFP isolation from HepG2 overexpressing the respective fusion proteins (Figure 2 and Figure S1). Therefore, macroH2A1 splicing isoforms, expressed as GFP-tag proteins, can be isolated separately through immunoprecipitation. This was the first step allowing us to analyze PTMs affecting macroH2A1.1 and/or macroH2A1.2 using mass spectrometry.

### 3.2. Identification and Quantification of Post-Translational Modifications (PTM) on GFP-Tagged macroH2A1.1 and macroH2A1.2

**Figure 3:**
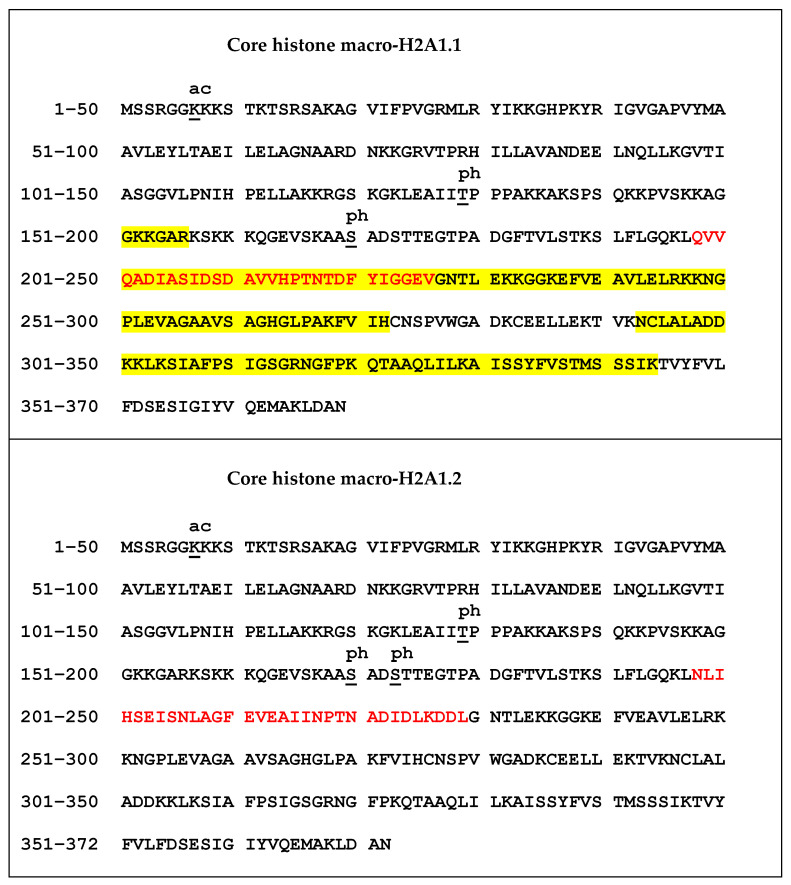
Characterization of macroH2A1.1 and macroH2A1.2 protein isoforms in HepG2 cells using LC-MS/MS. More than 75% of sequence coverage (indicated in yellow) was obtained for both isoforms using merged data from trypsin digestion combined with chemical derivatization of lysines. Only highly confident peptides identified using a fixed-value PSM validator node with Mascot parameters set to Rank 1, expectation value < 0.01, and ion score ≥ 30 were considered. Identified PTMs, including phosphorylation (ph) and acetylation (ac) are indicated. Quantified PTMs are colored in purple. Peptide carrying S173ph was identified only in a single replicate of macroH2A1.2 and could not have been quantified due to a very low intensity across the sample set. The amino acid sequence corresponding to the isoform-specific region is shown in red.

**Figure 4:**
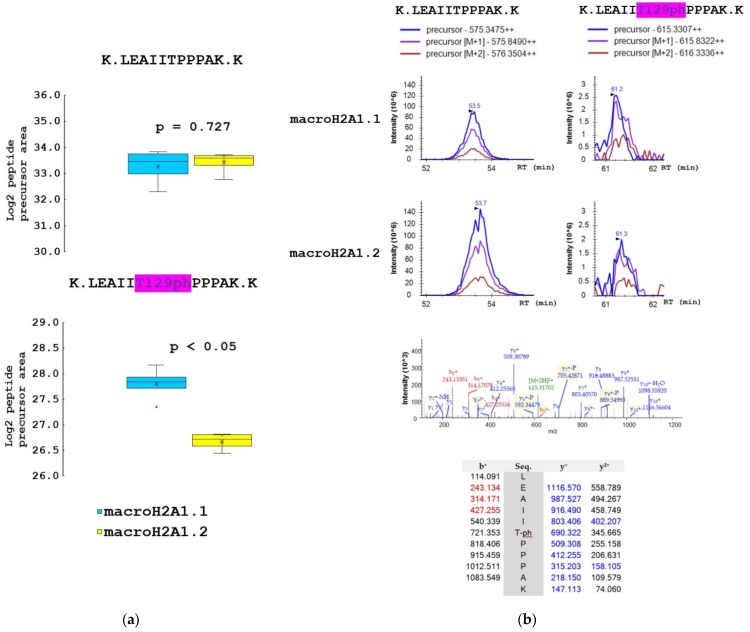
The proportion of T129ph mark on macroH2A1.1 and macroH2A1.2 protein isoforms in HepG2 cells. (**a**) Box-plots of the relative abundance of phosphopeptides and corresponding non-modified counterparts showing extremes, interquartile ranges, means, and medians (N = 4). Data were normalized to the sum of selected non-modified peptides to eliminate the effect of different expression levels of the two isoforms during quantitative analyses. Differences between samples in normalized peptide abundances ≥1.5-fold at *p* < 0.05 were considered as significant. (**b**) Representative MS extracted ion chromatograms of K.LEAIITPPPAK.K with (*m*/*z* 615.331) and without phosphorylation at T129 (*m*/*z* 575.348), showing their abundance in macroH2A1.1 and macroH2A1.2 isoforms. Representative MS/MS spectrum produced from the precursor ion of *m*/*z* 615.331 and fragments of y- and b-series with the *m*/*z* values are shown.

**Figure 5:**
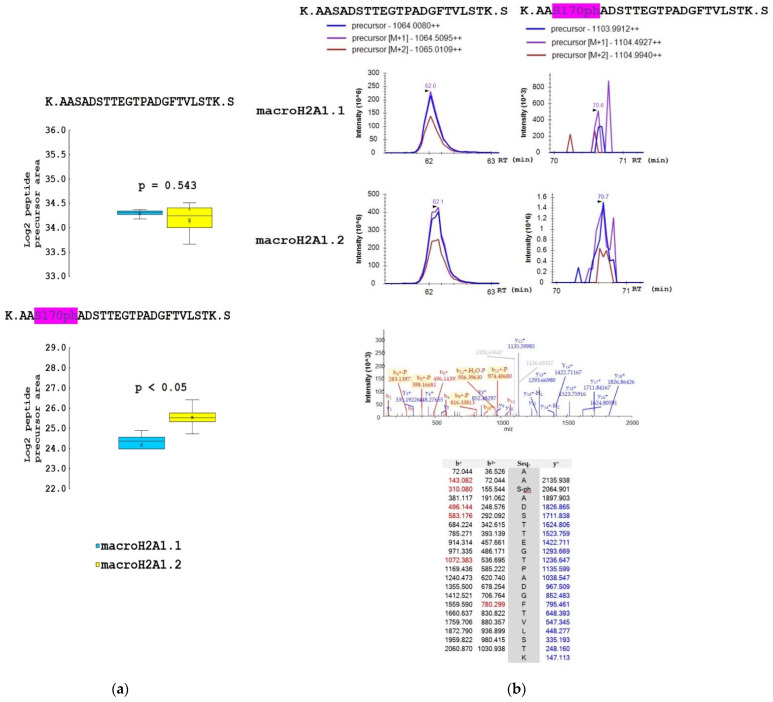
The proportion of S170ph mark on macroH2A1.1 and macroH2A1.2 protein isoforms in HepG2 cells. (**a**) Box-plots of the relative abundance of phosphopeptides and corresponding non-modified counterparts showing extremes, interquartile ranges, means, and medians (N = 4). Data were normalized to the sum of selected non-modified peptides to eliminate the effect of different expression levels of the two isoforms during quantitative analyses. Differences between samples in normalized peptide abundances ≥1.5-fold at *p* < 0.05 were considered as significant. (**b**) Representative MS extracted ion chromatograms of K.AASADSTTEGTPADGFTVLSTK.S with (*m*/*z* 1103.991) and without phosphorylation at S170 (*m*/*z* 1064.008), showing their abundance in macroH2A1.1 and macroH2A1.2 isoforms. Representative MS/MS spectrum produced from the precursor ion of *m*/*z* 1103.991 and fragments of y- and b-series with the *m*/*z* values are shown.

The samples for mass spectrometry analysis were enzymatically digested, either directly or after chemical derivatization of amine groups at lysine residues, to achieve higher sequence coverage of macroH2A1 isoforms (for details, see Section 2.2). Spectra of tryptic peptides obtained from both fractions were subjected to database search. The following PTMs were detected on macroH2A1 proteins with high confidence: K7ac, T129ph, S170ph, and S173ph. The merged database search results of both fractions are presented in Figure 3. Basically, the same set of PTMs was identified in both macroH2A1 isoforms. No PTM was detected within the isoform-specific region (198–229 AK). Phosphorylated peptides K.LEAIIT129phPPPAK.K and K.AAS170phADSTTEGTPADGFTVLSTK.S were identified in non-derivatized samples. The K.AASADS173phTTEGTPADGFTVLSTK.S peptide was identified only in a single replicate of macroH2A1.2; nevertheless, it documents that both S170 and S173 are phosphorylated in vivo (see the representative spectra of positional isomers in Supplementary Figure S2). While S170ph-peptide was intense enough for quantification, S173ph-counterpart could not be quantified due to a very low intensity across the sample set. Chemical derivatization enabled the identification of peptides with multiple lysines in the sequence, including R.GGK7acKKSTKTSR.S. While the levels of acetylated K7 histone marks were comparable between the two isoforms (Figure S3), significant quantitative differences were found in identified phosphorylated peptides. In particular, more than two-fold lower levels of LEAIIT129phPPPAK peptide together with a higher level of AAS170phADSTTEGTPADGFTVLSTK peptide were detected in macroH2A1.2 compared to macroH2A1.1, while the abundances of respective non-phosphorylated counterparts were comparable (Figure 4 and Figure 5).

### 3.3. Mapping PTM on UniProt Sequences of macroH2A1.1 and macroH2A1.2: Structural and Functional Considerations

#### 3.3.1. Isoform Structural Difference

**Figure 6:**
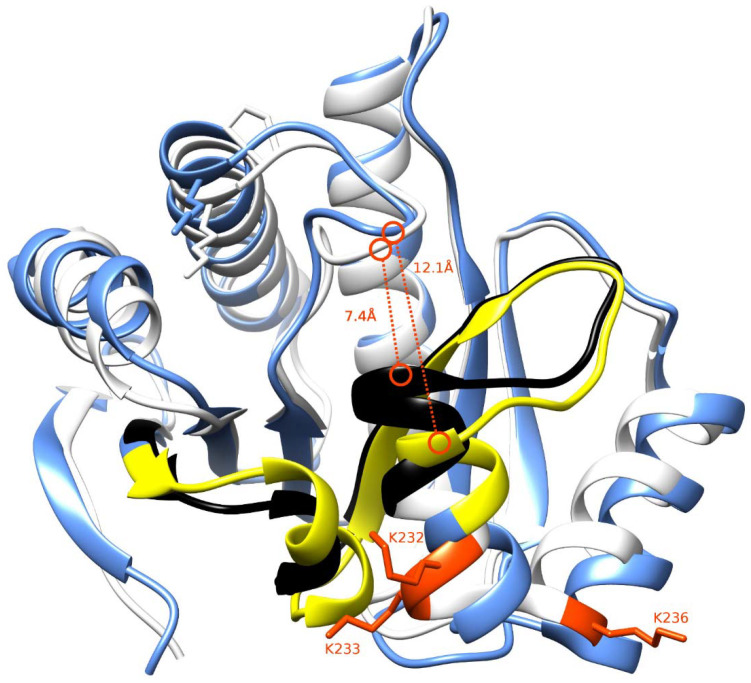
Three-dimensional structures of macroH2A1.1 and macroH2A1.2. The structures of macroH2A1.1 and macroH2A1.2 are drawn in white and light blue, respectively. The region showing sequence difference between the two proteins (198–229) has been highlighted in black for macroH2A1.1 and yellow for macroH2A1.2. The residues K232, K233, and K236 have been marked in red. The binding pocket’s width has been highlighted with a dashed red line reporting the measure in Ångstroms.

The two isoforms differ in the 30-residue long region spanning the residues 198–229. The macrodomain structures for macroH2A1.1 and macroH2A1.2 were structurally aligned to highlight similarities and differences. This region forms secondary structure elements directly involved in forming part of the ligand-binding pocket (Figure 6, regions colored in black and yellow for macroH2A1.1 and macroH2A1.2, respectively). As a consequence of this difference, macroH2A1.1 exhibits narrower access to the binding pocket (7.4 Å) than macroH2A1.2 (12.1 Å); this could also reflect differential ligand shape recognition and chemical features associated with binding affinities.

#### 3.3.2. PTM 3D Mapping

The phosphorylation events on T129, S170, and S173 map on the disordered region, which forms the linker between the histone H2A domain and the macro domain. This region is not covered by any crystallized structure currently deposited in the Protein Data Bank. Given the absence of structural data, we cannot confirm the involvement of phosphorylation sites in the regulation of NAD/ADP binding; however, we can observe that, since the first residues in the structures are G182 and D179 for, respectively, macroH2A1.1 and macroH2A1.2, S170 and S173 are in proximity of the region of interest.

#### 3.3.3. Regulation of Protein Interaction

Phosphorylation sites on disordered regions can function as docking points for recognition domain-mediated protein interactions [29]. Therefore, another possible function of the phosphorylation sites located in the linker region could be that of regulating the interactions between macroH2A and other proteins. The only structure where S170 and S173 are crystallized is 3hqh, where macroH2A is represented by a small peptide bound by SPOP (speckle-type POZ protein). SPOP is a component of the cullin-RING-based BCR (BTB-CUL3-RBX1) E3 ubiquitin-protein ligase complex that mediates the ubiquitination of target proteins, leading most often to their proteasomal degradation. This evidence connects these phosphorylations to a regulatory function.

There is no low-throughput data for the phosphorylations on T129, S170, and S173; however, they have been observed in different high-throughput experiments (PhosphoSitePlus [30]). Therefore, the responsible kinase can only be inferred computationally. We scanned the two 15-residue-long peptide sequences centered around S170 and S173 of macroH2A1.1 and macroH2A1.2 with AKID [22]. AKID linked both S170 and S173 with RPS6KC1 and CAMKK1; it then linked S170 with MINK1 and STK32A, while S173 was linked with casein kinase II subunit alpha and alpha’.

#### 3.3.4. NAD/ADP-Ribose Binding

**Figure 7:**
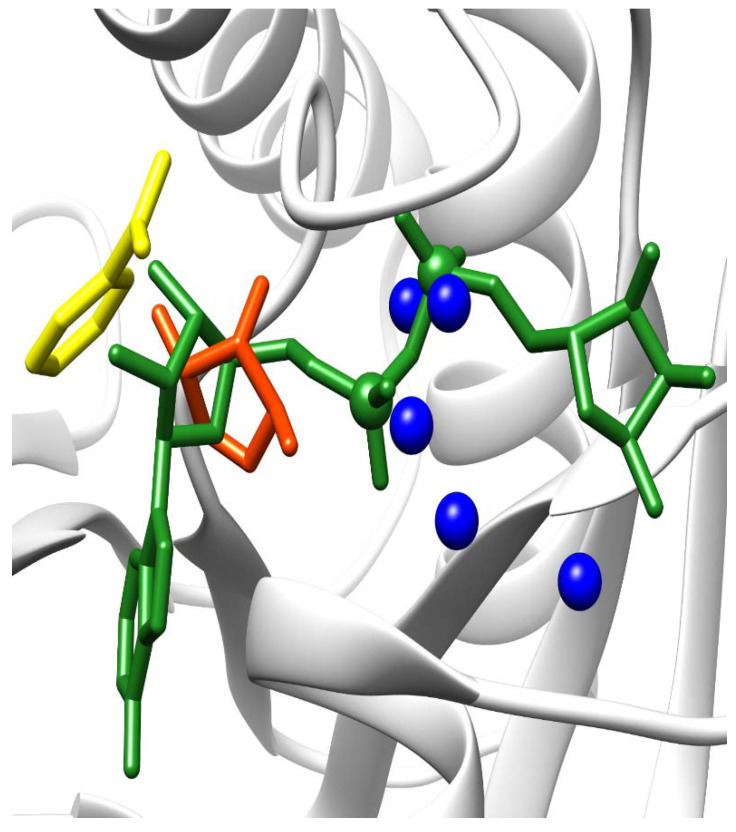
ADP-ribose binding site. The ADP-ribose molecule bound by macroH2A1.1 (PDB structure 3iid, colored in green) has been rototranslated onto the apo-form of macroH2A1.1 (PDB structure 1zr3, colored in white) after structural alignment. The nucleotide-binding site predicted by Nucleos is composed of one nucleobase-binding site (colored in yellow), a carbohydrate-binding site (colored in orange), and several putative phosphate-binding sites (colored in blue).

There is a structure of macroH2A1.1 binding ADP-ribose (PDB code 3iid), while there is no structure of macroH2A1.2 binding NAD/ADP-ribose. We used Nucleos [23,24] to predict nucleotide-binding sites on the surface of these proteins. Nucleos confirmed the binding site for ADP-ribose on macroH2A1.1 by predicting the binding site for a generic nucleotide di-phosphate (Figure 7); it is worth noticing that Nucleos has not been built to predict binding sites for ADP-ribose specifically. Moreover, Nucleos combined a nucleotide-binding site only in the close proximity of the real binding site and nowhere else. The nucleobase used by Nucleos for the prediction is nicotinamide (Figure 7, colored in yellow), which is a constituent of NAD. Therefore, the binding is confirmed by computational predictions by Nucleos and also by a crystal structure. Interestingly, Nucleos could not predict any nucleotide-binding sites on the surface of macroH2A1.2, for which no structures with the crystallized ligand have been released yet.

## 4. Discussion

In mammalian cells, chromatin is organized into the structural units of nucleosomes, composed of dimers of canonical histones H2A-H2B and H3-H4 [31]. Beyond DNA methylation and histone PTM (including acetylation, phosphorylation, methylation, and ubiquitination), one of the most relevant and perhaps underappreciated epigenetic modifications is the substitution of canonical histones with non-canonical ones: histone variants, which satisfy important functions that cannot be performed by canonical histones [1,32]. In this study, we focused on macroH2A1, an H2A variant, which is a transcriptional modulator that can either repress transcription or activate it in response to growth signals in somatic cells, stem cells, and cancer cells [12]. A major open question in the field is how the two exon splicing isoforms of macroH2A1, macroH2A1.1, and macroH2A1.2, can play such remarkably different roles in the regulation of stemness and tumorigenicity in animal models and in humans, suggesting that macroH2A1 isoforms constitute distinct “locks” at critical pluripotency and proliferative genomic sites [8,10,33,34,35,36,37]. MacroH2A1.1 and macroH2A1.2 diversity relies on their different primary structure, influencing protein folding and resulting in different accessibility of their C-terminal macrodomain [12]. Alignment of the macrodomain regions of the two isoforms results in a non-identical 30-residue-long region spanning the residues 198–229 (Figure 1 and Figure 6) [10]. As a consequence, as also highlighted by Nucleos and by an X-ray-derived 3D structure, macroH2A1.1 is the only splicing variant able to bind ADP-ribose moieties (Figure 7). Our aim here was to investigate whether the structural/functional differences between the two splicing isoforms could be predicted, at least in part, by the characterization of common and different PTMs in their structures. We thus overexpressed individually macroH2A1 splicing isoforms as GFP-tag fusion proteins in human HepG2 cells [17]. In 2006, it was reported that the tagging of the macroH2A1 splicing isoforms with GFP does not affect its colocalization and function, thus representing a valid tool to analyze PTMs [14]. In our study, we isolated macroH2A1.1/1.2-GFP fusion proteins using GFP-Trap®, which is a nanobody-based reagent launched in 2008 and is now the gold standard for immunoprecipitation of GFP fusion proteins. We identified K7 as a new acetylation site in both macroH2A1 isoforms in HepG2 cells. Methylated lysines at positions 18, 123, and 239 in macroH2A1.2, previously identified by Chu et al. (2006) in embryonic kidney cells [14], were not found in HepG2 cells in our study. We have not identified S137ph, previously reported in HEK293 cells [14]. On the other hand, T129ph was found in all mass spectrometry-based executed studies aimed at macroH2A characterization, showing less cell-specificity, thus representing a global PTM feature [14]. Even though mass spectrometry analyses did not reveal post-translationally modified amino acids in macroH2A1.1 binding pocket, this possibility cannot be excluded, as it represents a lysine-rich region (i.e., K232, K233, and K236). Nevertheless, there is an increasing evidence that macroH2A1.1/1.2 C-terminal domain is affected by PTMs on vicinal sites. Next to the T129ph, S170 and S173 phosphorylation sites on the linker domain flanking the C-terminal domain for both macroH2A1 splicing isoforms were detected. Importantly, we found that T129 presented higher levels of phosphorylation in macroH2A1.1 compared to macroH2A1.2 (Figure 4) while, on the contrary, S170 phosphorylation levels were enhanced in macroH2A1.2 (Figure 5). We thus speculate that different phosphorylation levels of these two residues may affect the folding of macroH2A1 isoforms, also contributing to macroH2A1.1 ability to bind ADP-ribose. In addition, this region is phosphorylated by Haspin protein kinase in HEK293 cells, relating this PTM with the macroH2A1-DNA interaction [38,39]. Our computation analysis showed that other kinases may be involved in S170 phosphorylation, despite none of them has been validated experimentally yet. Interestingly, phosphorylation sites on disordered regions, such as the macroH2A1 linker domain, may play an important role for recognition domain-mediated protein interactions [29,40]; moreover, the linker region stabilizes heterochromatin architecture [41,42].

## 5. Conclusions

Taken together, our results suggest how the different PTMs on the two macroH2A1 splicing isoforms may be responsible for their distinct features and for their distribution in the cell. Particularly, extreme structural flexibility of disordered regions determined by the levels of PTMs presented at multiple interaction sites emerges as a crucial regulatory element in macroH2A1 variants, enabling dynamic interaction with various partner proteins.

## Data Availability

The mass spectrometry proteomics data have been deposited to the ProteomeXchange Consortium via the PRIDE [19] partner repository with data set identifier PXD022968.

## Supplementary Materials

The following are available online at https://www.mdpi.com/article/10.3390/biology10070659/s1, Figure S1: Full western blot images showing macroH2A1.1-GFP and macroH2A1.2-GFP isolation. Figure S2: The identification of S173ph mark on macroH2A1.2 in HepG2 cells, Figure S3: The proportion of K7ac mark on macroH2A1.1 and macroH2A1.2 protein isoforms in HepG2 cells.
