# Supplementary figures and images for: Phosphorylation within Intrinsic Disordered Region Discriminates Histone Variant macroH2A1 Splicing Isoforms—macroH2A1.1 and macroH2A1.2
Source: Biology (Basel). 2021 Jul 13;10(7):659. doi: 10.3390/biology10070659 (PMC8301376; doi:10.3390/biology10070659)

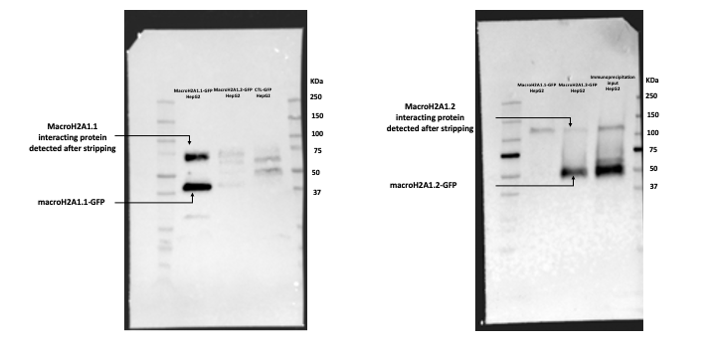

Supplement: Supplementary file 1 [file biology-10-00659-s001.zip › biology-1284247-supplementary.tiff]
